# Essential steps in the development, implementation, evaluation and quality assurance of the written part of the Swiss federal licensing examination for human medicine

**DOI:** 10.3205/zma001564

**Published:** 2022-09-15

**Authors:** Tina Schurter, Monica Escher, David Gachoud, Piotr Bednarski, Balthasar Hug, Roger Kropf, Juliane Meng-Hentschel, Benjamin König, Christine Beyeler, Sissel Guttormsen, Sören Huwendiek

**Affiliations:** 1 University of Bern, Institute for Medical Education, Department for Assessment and Evaluation, Bern, Switzerland; 2 University of Geneva, Medical Faculty, Geneva, Switzerland; 3 University of Lausanne, Medical Faculty, Lausanne, Switzerland; 4 University of Fribourg, Medical Faculty, Fribourg, Switzerland; 5 University of Bern, Medical Faculty, Bern, Switzerland; 6 University of Basel, Medical Faculty, Basel, Switzerland; 7 University of Lucerne, Medical Faculty, Lucerne, Switzerland; 8 University of Zurich, Medical Faculty, Zurich, Switzerland

**Keywords:** national final examination, licensing examination, summative assessment, multiple choice

## Abstract

**Purpose:**

This report describes the essential steps in the development, implementation, evaluation and quality assurance of the written part of the Swiss Federal Licensing Examination for Human Medicine (FLE) and the insights gained since its introduction in 2011.

**Methods:**

Based on existing scientific evidence, international expertise, and experience gained from previous examinations, the FLE is developed by experts from all five medical faculties in Switzerland with the support of the Institute for Medical Education and is held simultaneously at five locations. The exam organisers document and review every examination held and continuously optimise the processes; they have summarised the results in this report.

**Results:**

The essential steps comprise the development, revision and translation of questions; construction of the exam and production of materials; candidate preparation; implementation and analysis. The quality assurance measures consist of guideline coherence in the development of the questions and implementation of the exam, revision processes, construction of the exam based on the national blueprint, multiphase review of the translations and exam material, and statistical analysis of the exam and the comments from candidates. The intensive collaboration, especially on the part of representatives from all the participating faculties and a central coordination unit, which provides methodological support throughout and oversees the analysis of the exam, has proven successful. Successfully completed examinations and reliable results in the eleven examinations so far implemented represent the outcomes of the quality assurance measures. Significant insights in recent years are the importance of appreciating the work of those involved and the central organisation of exam development, thus ensuring the long-term success of the process.

**Conclusion:**

Common guidelines and workshops, quality assurance measures accompanied by the continuous improvement of all processes, and appreciation of everyone involved, are essential to carrying out such an examination at a high-quality level in the long term.

## Zusammenfassung

**Zielsetzung:**

Dieser Bericht beschreibt die zentralen Schritte der Erstellung, Durchführung, Auswertung und Qualitätssicherung des schriftlichen Teils der eidgenössischen Prüfung Humanmedizin (Schweizer Staatsexamen) sowie die seit deren Einführung 2011 gewonnenen Erkenntnisse.

**Methodik:**

Die eidgenössische Prüfung wird auf Basis vorhandener wissenschaftlicher Evidenz, internationaler Expertise sowie Erfahrungen aus vorhergehenden Prüfungen durch Experten aller fünf Medizinischen Fakultäten der Schweiz mit Unterstützung des Instituts für medizinische Lehre erstellt und gleichzeitig an fünf Standorten durchgeführt. Die Prüfungsorganisatoren dokumentieren und reflektieren jede Prüfungsdurchführung, optimieren die Prozesse kontinuierlich und haben die Ergebnisse in diesem Bericht zusammengefasst.

**Ergebnisse:**

Die zentralen Schritte umfassen: Fragenerstellung, -revision, -übersetzung; Prüfungszusammenstellung und Materialherstellung; Kandidatenvorbereitung; Prüfungsdurchführung und -auswertung. Die Qualitätssicherungsmassnahmen bestehen aus Richtlinienkohärenz bei der Fragenerstellung und Prüfungsdurchführung, Revisionsprozessen, Prüfungszusammenstellung auf Basis des nationalen Blueprints, mehrstufiger Überprüfung der Übersetzungen und des Prüfungsmaterials sowie der statistischen Auswertung der Prüfung und der Kandidatenkommentare. Bewährt hat sich dabei die enge Zusammenarbeit insbesondere von Vertretern aller beteiligten Fakultäten sowie einer zentralen Koordinationsstelle, die sämtliche Schritte methodisch begleitet und sich um die Auswertung der Prüfung kümmert. Die Endergebnisse der Qualitätssicherungsmassnahmen sind erfolgreiche Prüfungsdurchführungen und reliable Ergebnisse in den bisher elf so durchgeführten Prüfungen. Wichtige Erkenntnisse der vergangenen Jahre sind die Bedeutung der Wertschätzung der Arbeit der Beteiligten und die zentrale Organisation der Prüfungserstellung, um den Prozess langfristig erfolgreich durchzuführen.

**Schlussfolgerung:**

Um eine solche Prüfung langfristig mit hoher Qualität durchzuführen, sind gemeinsame Richtlinien und Workshops, Qualitätssicherungsmassnahmen mit fortlaufender Verbesserung aller Prozesse sowie die Wertschätzung der Beteiligten zentral.

## 1. Introduction

As a result of the Federal Act of 23 June 2006 on University Medical Professions (Medical Professions Act, LPMed), which came into effect on 1 September 2007, the Swiss Federal Licensing Examination for Human Medicine (FLE) had to be restructured and centrally organised. Following a preparation period, accordingly, a national (federal) licensing examination was introduced in 2011 which is to be taken after completion of medical studies at master’s level based on current learning objectives (2011-2020: *Swiss Catalogue of Learning Objectives for Undergraduate Medical Training (SCLO)* [1], since 2021 *Principal Relevant Objectives and Framework for Integrative Learning and Education in Switzerland (PROFILES)* [http://www.profilesmed.ch/]). On successful completion, the candidates may practise their medical profession under supervision and commence their further medical training. The examination consists of two parts [2]: a multiple-choice exam (MC) and a structured practical clinical exam involving standardised patients (clinical skills (CS)) [3]. This has replaced the former final examinations which were done in several specialties. The challenge in developing this new examination was to design – in the most efficient way possible and by involving the five existing medical faculties in two language regions – a common cross-disciplinary, application-orientated exam weighted according to a multidimensional blueprint that would guarantee eligibility for further training at the national level, meet international quality standards, and be legally robust.

There is considerable interest in national licensing examinations [4], [5], [6]. Other countries are considering their introduction (Great Britain [6] and Norway) or looking to harmonise the practical exam in situations where a common written licensing examination already exists (Germany [7]). There are few publications, however, on experience and insights from the joint development of cross-faculty or national written examinations [8]. Edwards et al. [9] demonstrated, in the Australian context, that the following factors in particular foster successful collaboration on exam development: *committed group (e.g. medical schools, stakeholders), funding (e.g. development grant), engagement (team meetings to build vision), products (e.g. framework, assessment development), ownership (e.g. open to all medical schools)*. In addition, articles have been published on specific aspects such as automated question generation for the national exam in Canada [8], [10] or the use of computer-based [11], [12] or adaptive [13] tests in such a framework. 

Overall, there are only few publications on how to develop a high-quality written exam designed specifically as a national licensing examination. Considering the additional aspect of bilingual implementation, even fewer reports can be found. 

This report follows up on previous publications concerning the Swiss FLE [2], [3]. It addresses the essential steps, quality assurance measures and insights from the perspective of the organisers of the written part of the FLE following the eleven exam cycles that have taken place to date and offers interested parties the chance to compare and obtain reference points for similar projects of their own.

## 2. Methods

### Setting

The MC exam is an examination with two parts, each of which consists of 150 MC questions to be completed in 4.5 hours. It is closely coordinated and takes place simultaneously at all five medical faculties. For general information on this examination, please refer to the previously published overview article [2]. In addition to subject matter experts from the hospitals, representatives from general medical practice, methodology experts (expertise in question development, revision and evaluation), IT specialists, administrators, professional translators, representatives from the Federal Office of Public Health (FOPH), and the federal board of examiners, are involved in the examination process. The board of examiners is the most important decision-making body and is made up of vice-deans for teaching from the medical faculties and representatives from general medical practice, continuing education, and the FOPH. The methodology experts make up the national MC working group with representatives from the faculties and the Institute for Medical Education (IML). Except for recruiting question writers and implementing the examination at the faculties, the IML is responsible for coordination. 

#### Development of this project report

This project report is based on the experience gained from the eleven written federal examinations held since 2011. After each examination season, the recent exam along with quality assurance and quality indices are reviewed and documented to ensure continuation of what has proven effective and to identify potential areas for improvement. The essential development, implementation, evaluation and quality assurance steps, along with the insights, are collated by the exam organisers in an iterative process. 

## 3. Results

**Figure 1:**
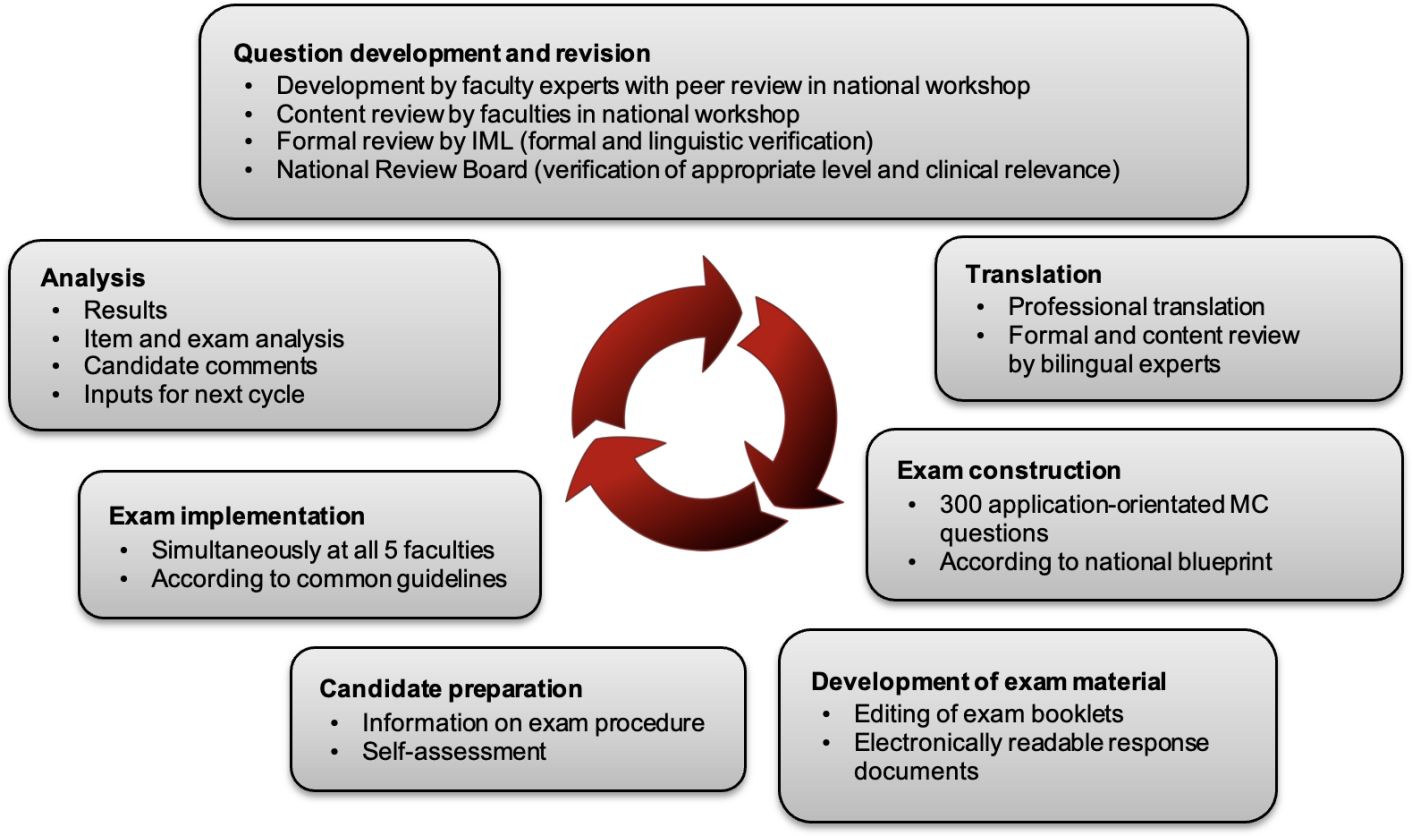
Essential steps in the examination process

The examination process is explained in detail below. Figure 1 (Fig. 1) illustrates the most important steps of the process; table 1 (Tab. 1) provides a summary of quality assurance measures and insights. 

### 3.1. Question development and revision

Around 60 experts nominated by the faculties write draft questions that are discussed and, if necessary, revised in author groups (peer review) during the first day of a two-day workshop. On the second day of the workshop, the new questions are reviewed for a second time and finalised in editorial groups consisting of at least one representative from each faculty and representatives from different medical disciplines. Any old questions that were created five years previously or were conspicuous in the most recent examination due to statistical deviations or candidate comments are reviewed for their relevance and revised by the same editorial groups. Following a centralised formal and linguistic review of the new questions by methodology experts at the IML, the questions are examined by a national review board (consisting of physicians from different disciplines of all faculties who are well versed in the guidelines on question development for this examination and also write questions themselves) to verify their suitability (level of difficulty) and clinical relevance before they are approved in the database for construction of the next exam. The questions are managed using software developed at the IML [https://www.iml.unibe.ch/themen/uebersichten/projekte].

#### Quality assurance

The requirements for the question writers cover the content-related basics (learning objectives and the resulting content blueprint), information on the types of questions, the specified standard structure for case vignettes, and formulation principles according to Krebs [14]. Preference is given to questions with case vignettes and thus application-orientated questions involving, among other things, diagnostic or therapeutic decisions. 

The guidelines published by the Medical Professions Commission (MEBEKO) state the following regarding the relevance of the questions to practical application [15]: “The clinical knowledge exam (written) tests the interdisciplinary, application-orientated knowledge of the entire spectrum of problems in human medicine across the disciplines” and “The questions should relate to a specific problem, wherever possible, that is presented in a case or problem vignette”. This is specified in detail, with examples, in the internal instructions or training documents for the authors. Each question should be as authentic as possible, i.e. reflect a doctor’s daily work. To this end, it should refer wherever possible to a specific case in which a patient is described (age, gender, setting, reason for consultation, medical history, status/findings, possible results from diagnostic tests, perhaps initial treatment, follow-up results etc.) and a specific question must be answered, e.g. “What is the most likely diagnosis?", “What is the most useful therapeutic approach?”. In addition, every question should be clinically relevant.

With respect to the question types, MEBEKO provides the following definition [15]: “Selection of the only correct or best from 3-5 offered choice answers (type A, positively or negatively formulated) and fourfold decision right/wrong (type Kprim).”

The questions to be written are determined based on the learning objectives and blueprint (see table 2 (Tab. 2)). The blueprint also defines the percentage distribution of the questions in terms of content. All authors contribute also to teaching at their faculties and developing questions for the faculty exams. As part of the workshop, they receive informational material beforehand concerning the development of draft questions that will familiarise them with the FLE standards. The involvement of all faculties in every revision step (including deletion of questions in exceptional cases) facilitates the development of a nationally valid examination accepted by all training institutes. 

##### Insights

The survey of the workshop participants revealed that the format used since 2017 for developing questions (workshop without daily distractions, exchange with expert colleagues throughout Switzerland) is regarded as highly motivating [16], unlike the decentralised process of writing questions alone that was in place until 2016. The workshop format also increases the output of new questions of higher quality (less need for revision). This concentrated two-day effort is facilitated by the secure web-based question database mentioned above. 

#### 3.2. Translation

Depending on the author, the questions are formulated in German or French. Following the revision process, they are translated into the other language, respectively, by professional translators. The translations undergo formal and linguistic review by methodology experts, while medical specialists check the content.

##### Quality assurance

The quality of the translations is assured by multiphase review. After the examination, candidate comments and the patterns of responses to the questions are analysed to identify any translation errors or technical ambiguities. Questions containing translation errors are excluded from the evaluation, as are other questions with possible formal deficiencies.

##### Insights

Isolated translation issues have been addressed in some candidate comments. Hence, in addition to the existing control process, all questions now undergo a review by a medical professional in their native language. 

Having adapted and introduced additional control steps in the translation review process, fewer questions have had to be rejected due to poor translations.

#### 3.3. Exam construction

A total of 300 questions are selected from the question database for each examination in line with the nationally applicable blueprint (for the first two dimensions, see table 2 (Tab. 2)), which was developed on the basis of the learning objectives [1], [http://www.profilesmed.ch/]. The current blueprint consists of three main dimensions:

- Dimension 1: *Situations as starting points, *
- Dimension 2: *Medical tasks*, and
- Dimension 3: *General objectives *(focus on medical expert). *Type of condition (acute, subacute, chronic)* and *setting (ambulatory practice, hospital, nursing home for elderly people, other)* are further dimensions of the blueprint.

To prevent the strategic omission of individual fields of knowledge during study, the percentage target values are not published and thus are not specified in the table. At least 20% of the questions that have proven effective in previous examinations are used. The compatibility of learning objectives and exam questions is guaranteed by the clear definitions provided in the examination regulations, and content-wise by the blueprint and the criteria for the types of questions selected. 

##### Quality assurance

The representative distribution of the content is ensured by the blueprint and by the candidate survey following the exam using a questionnaire with open-ended and closed-ended questions. By tagging the questions in the question database, compliance with the blueprint is supported.

##### Insights

Analysis of the questionnaire revealed that it is important to many candidates to cover the studied subjects as comprehensively as possible (comments on open-ended questions in the questionnaire) and to have questions that in terms of content reflect the learning objectives (with a median of 4 on a scale of 1 (do not agree at all) to 5 (completely agree), closed-ended question in the questionnaire). Compliance with the blueprint and broad coverage is only possible thanks to detailed tagging of the questions.

#### 3.4. Development of exam material

The templates for the exam booklets are generated from the question database and printed before being sent to the faculties. The subjects covered by the questions are divided equally between the two exam booklets; the length of the text in each booklet is identical.

##### Quality assurance

The quality of the exam booklets is assured by means of multiphase review involving verification of the content and formal aspects (form and language) by experts.

##### Insights

Despite the preceding revision process during question development and translation, anomalies are still occasionally detected when reviewing the print proofs. These are checked and corrected by physicians during a content review phase.

#### 3.5. Candidate preparation

Candidates can obtain information from the website of the Federal Office of Public Health and faculty informational events concerning the examination procedure [15] and receive nationally standardised documents from the person responsible at the respective site. In addition, around 300 representative sample questions are made available online in a self-assessment tool [https://www.iml.unibe.ch/angebote/assessment/pruefungsdienstleistungen/self-assessment]. 

##### Quality assurance

The preparatory information is reviewed every year and adapted, as necessary. 

##### Insights

Feedback from the candidates in the questionnaire and argumentation in objections to the exam result have shown that not all candidates actively study the information provided prior to the exam. Hence, this information is also sent directly to each candidate in advance.

#### 3.6. Exam implementation

The exam takes place on two days at the same time (4.5 hours each) at five faculties. The exam is taken not only by successful graduates of master’s programmes in Switzerland but also by candidates holding unrecognised foreign medical qualifications issued outside of the EU/EFTA.

##### Quality assurance

Common guidelines on exam implementation regulate the conditions of the examination premises, aids permitted, and instructions to be read out during the exam, for example. 

##### Insights

Some candidates find it difficult to absorb information or instructions on the day of the exam. Hence, they additionally receive all the information in advance. Suggestions entered in the questionnaire by the candidates help to continually improve the procedure.

#### 3.7. Evaluation and results

An item analysis is performed for every exam question set (assessment of item measurement properties). Difficulty and discrimination are primarily assessed; these remain visible in the question pool. If a question is reused, it can thus be compared across different exam years. The aim is to achieve a minimum of 0.2 as the item discrimination index (r), and 50-90% for P as the degree of difficulty. The multiple true/false questions of the Kprim type are analysed using the half-point method developed at the IML [17].

The exam results and candidate comments on questions that appear to be flawed are initially analysed by methodology experts. Conspicuous questions are discussed with several clinical experts and excluded from the analysis if formal or content-related deficiencies are detected: for example, if a question that is too difficult is found to be of “specialist” level, or a previously overlooked formal error is identified when the r value is too low. 

The passing score is based on two internationally recognised standard-setting methods (Angoff and Hofstee [18], [19], [20]) and an analysis based on the Rasch model [21]. The Rasch model is the simplest model of the item response theory (IRT) in which the item difficulties and the ability of the candidates can be estimated as a means of explaining the exam results. By using items with a difficulty already observed in previous examinations that are therefore known (referred to as anchor items), the model permits the pass requirement to be maintained at a consistent level over the years even if the difficulty of the exam varies each year. Put simply: a comparison between anchor items and the rest of the examination means the examination difficulty can be judged against the previous years, and the passing score can thus be adapted accordingly. The pass grade is therefore set by the board of examiners using the standard-setting methods and the Rasch model.

##### Quality assurance

The final decision concerning the passing score is made by the national board of examiners. Formal or content-related deficiencies in exam questions discovered during the analysis are reported to the authors and the review board so that the next round of question development can be optimised accordingly. Questions with poor difficulty and discrimination scores and content-related deficiencies are not reused. All important analysis steps are carried out under the four-eyes principle: prior to publication, the most important results are reviewed by a second individual based on a checklist. For example: Was the passing score ultimately agreed by the board of examiners and recorded in the meeting minutes actually applied in the results lists, the charts in the analysis report, and in the letters to the candidates? Are the answers on the answer sheets of candidates who have failed actually consistent with the electronically recorded answers considered in the results calculation? The analyses represent an essential final step in assuring the quality of the entire examination process; further details are provided in Chapter 4 of [14]. 

##### Insights

Continuously optimised quality assurance measures throughout the examination process, including reporting of deficiencies discovered in the exam questions to the authors and the review board, mean that the number of questions having to be excluded from the analysis due to formal or content-related deficiencies has continually decreased over the years (approx. 40 from the first (13.3%) and about 15 (5%) from the most recent examination). The results from the eleven examinations implemented to date revealed thoroughly reliable exam results (Cronbach’s alpha average 0.90, range 0.87-0.91). 

**Figure 2:**
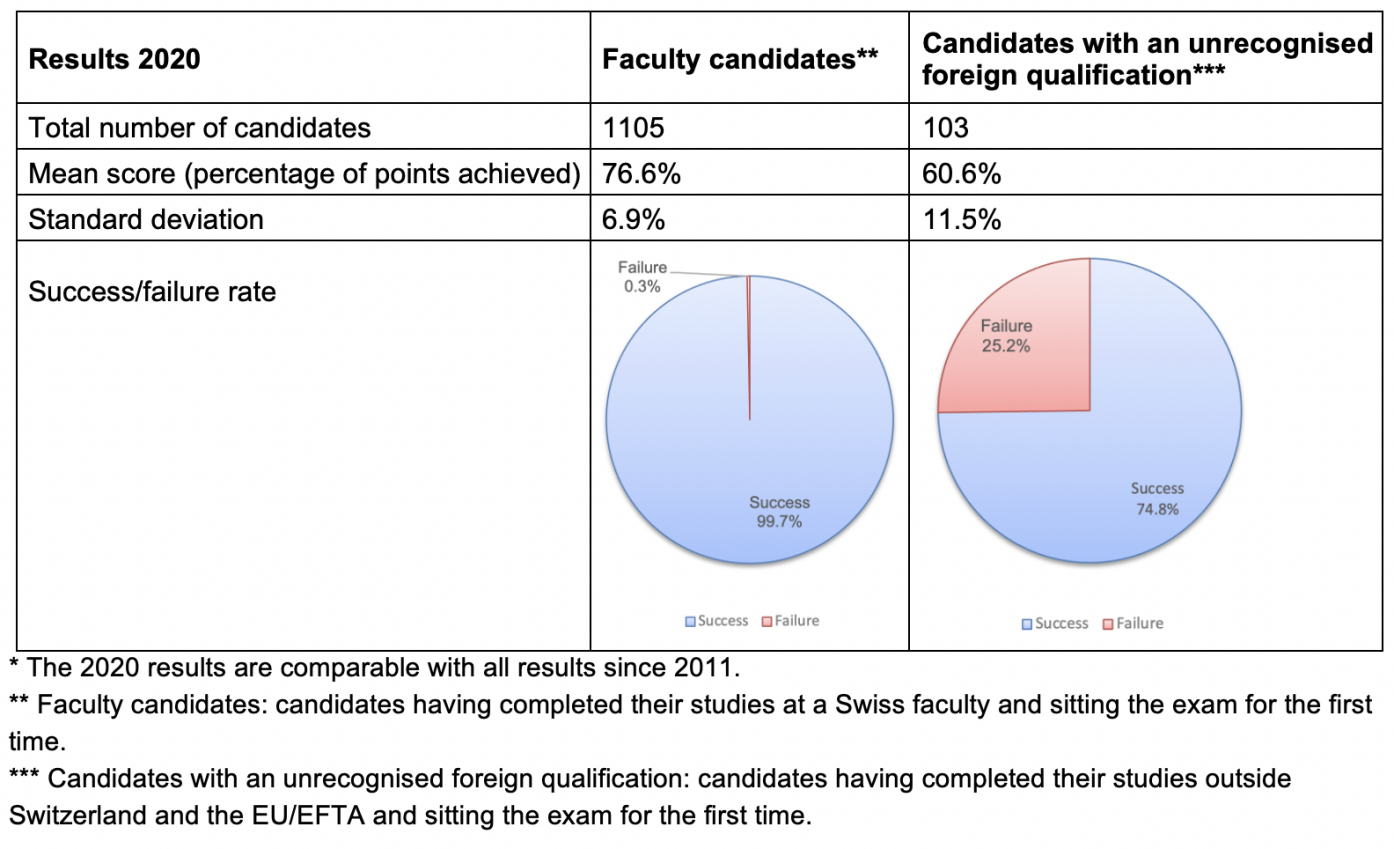
Exam results 2020*

Figure 2 (Fig. 2) lists the results from the examination 2020 as an example. The success rates of the candidates who had completed their studies at a Swiss faculty (referred to as “faculty candidates”) were consistently high, at 99.5%, whereas those of candidates with an unrecognised foreign medical qualification were much lower. This could be interpreted in such a way that Swiss candidates have also been taught using the Swiss Catalogue of Learning Objectives and thus there is a good alignment between their curriculum and examination, whereas this is not the case with candidates from abroad. Another explanation could be the linguistic challenges faced by foreign candidates or that their qualification has often been obtained several years previously and their level of knowledge is no longer the same.

## 4. Discussion

Important quality assurance measures entail, among others, the common guidelines on the development of questions and implementation of the examination, the preparation of questions in national workshops, the multiphase revision process involving all faculties, construction of the examination based on the national blueprint, and multiphase review of the translations and the exam material. Analysis of the examination, including analysis of candidate comments, is a final, significant quality assurance step in the entire examination process. 

Though Edwards et al. [9] also described some of these processes, including success factors, we can add insights into aspects of question development in national workshops, bilingualism, and analysis. Furthermore, the inclusion of all faculties in the design of the blueprint and in all phases of question development, revision and implementation leads to greater acceptance among the faculties and increases the credibility of the results achieved by the candidates.

The correlation between the written exam results and the results of the practical exam (average since 2011: 0.56; range: 0.48-0.65) suggests that the questions offer a degree of practical relevance given that the tasks set in the OSCE are practice orientated. We believe that, in the future, it would be desirable to verify that those graduates passing this entire exam (written and practical) are also providing their patients with appropriate care as competent doctors. This association is evident from similarly standardised, quality-assured examinations abroad [20], [21], [22], [23], [24].

It is important to note that this article addresses the multiple-choice part only, which becomes a complete examination [2] when supplemented by the previously published clinical skills part [3]. The publication on the clinical skills part of the examination [3] describes how the consistent implementation of the principles of action research contributes to the successful ongoing development of the practical exam. Furthermore, it describes how the centrally coordinated, collaborative iterative process involving experts from all faculties contributes significantly to the quality of the final clinical skills examination (FLE CS) [3]. A qualitative study among participating examination experts and deans of studies also revealed positive implications with respect to the overall examination, such as the intensified and positive collaboration between the faculties and the increased introduction of practical courses [24]. 

The differences in the pass rates of the graduates of Swiss faculties versus candidates with foreign medical qualifications not recognised in Switzerland can also be understood as supporting the validity of this exam. Such differences in national examinations have also been identified in other countries [25]. One reason could be that the training provided in Switzerland is geared towards the national learning objectives [1], [http://www.profilesmed.ch/] such that candidates from Switzerland are better prepared for the examination. Another explanation may be that most candidates with unrecognised foreign medical qualifications have pursued their further training in one discipline and are no longer so familiar with the general exam content. Appreciation for the work of those involved and the central organisation of exam development are two of the major insights gained in recent years that are essential to the long-term success of the process. The appreciation of those involved in medical teaching is a key aspect, as the clinical and scientific activities are frequently given a higher weighting when they are more conducive to individual careers. It can therefore prove difficult to find suitable individuals for teaching positions and assessment tasks, in particular. This appears to be a common challenge, as (inter)national surveys have shown [26], [27]. According to our experience, by holding national workshops in a seminar hotel to develop the questions and recognising these efforts as a teaching activity – rather than several smaller workshops or working individually at home – participants gain greater appreciation and a strategy is ultimately found that delivers better quality and is more economical [16]. 

The strengths of this report lie in the fact that it is based on the many years of practical experience gained by the various participants in the development, implementation and evaluation of this national examination, which is currently held in two national languages at five locations. The exam results have proved thoroughly reliable in the last eleven years, which is very much due of course also to the large number of exam questions.

One weakness is that this report is based on experience that has not been subjected specifically to scientific study. However, the insights discussed are based on a continuous optimisation process that is thoroughly documented, traceable, and comprehensible, and has been summarised by the authors for this report. 

Looking ahead, it can be said that the development of this examination will steadily continue. Since 2021, the examination has been subject to the new “PROFILES” set of learning objectives [http://www.profilesmed.ch/]. The blueprint is now based on this framework, accordingly. The structure of the blueprint, in particular, has therefore been simplified. There are fewer dimensions, for instance, as the previous dimensions of “age” and “gender” and part of the old “medical action” dimension are already considered under the “situations as starting points” of PROFILES, and could be deleted or shortened accordingly. On the other hand, the differentiation under “setting” (practice, hospital, residential care, other) is now greater than in the SCLO (inpatient, outpatient), as the precise setting of the described case is often critical to eliciting the correct answer. Furthermore, the previous dimension of “problems as starting points” has become “situations as starting points” in PROFILES, with physiological situations also listed (e.g. “process and basic care of pregnancy”, “well-baby and well-child visit”), and disciplines such as “emergency” and “palliative care” occupying a more prominent position. It has also been decided that, as of 2022, the written examination will be implemented digitally at all sites using tablet computers. Consequently, new question types can be used in the future that will permit in large part the automated analysis of free texts based on long menus [28]; videos can also be included, moreover.

## 5. Conclusion

Common guidelines, joint workshops, quality assurance measures with ongoing optimisation of all processes, and appreciation for all involved, are essential to carrying out such an examination at a high-quality level in the long term. 

## Acknowledgements

A national examination can only be achieved with the support of countless dedicated individuals. Our special thanks go to the participants of the workshops and review boards, and those responsible at the sites. We also thank the board of examiners and the Federal Office of Public Health for their valuable input and support.

## Competing interests

The authors declare that they have no competing interests. 

## Figures and Tables

**Table 1:**
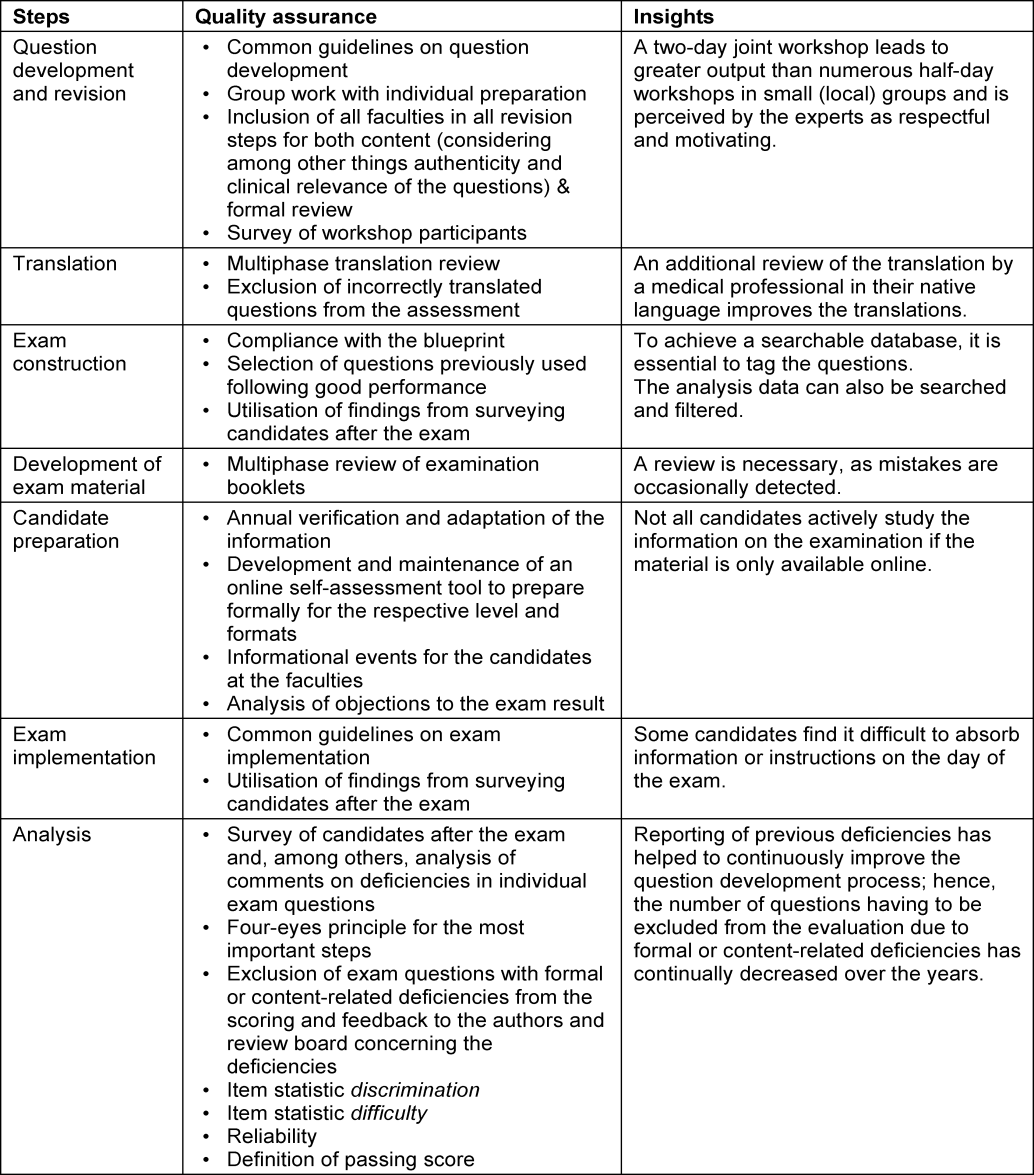
Summary of quality assurance and insights

**Table 2:**
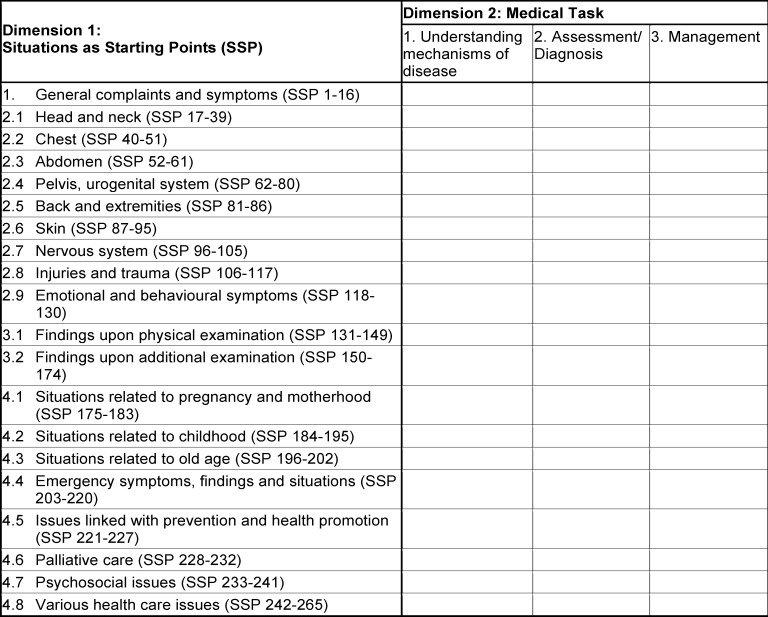
Current blueprint (BP) (according to PROFILES, valid as of 2021) for the written part of the Swiss Federal Licensing Examination for Human Medicine – main dimensions 1 and 2
